# Complex interactions of cellular players in chronic Graft-versus-Host Disease

**DOI:** 10.3389/fimmu.2023.1199422

**Published:** 2023-06-26

**Authors:** Laura Marie Gail, Kimberly Julia Schell, Piotr Łacina, Johanna Strobl, Steven J. Bolton, Emilie Steinbakk Ulriksen, Katarzyna Bogunia-Kubik, Hildegard Greinix, Rachel Emily Crossland, Marit Inngjerdingen, Georg Stary

**Affiliations:** ^1^ Department of Dermatology, Medical University of Vienna, Vienna, Austria; ^2^ CeMM Research Center for Molecular Medicine of the Austrian Academy of Sciences, Vienna, Austria; ^3^ Translational and Clinical Research Institute, Faculty of Medical Sciences, Newcastle University, Newcastle upon Tyne, United Kingdom; ^4^ Laboratory of Clinical Immunogenetics and Pharmacogenetics, Hirszfeld Institute of Immunology and Experimental Therapy, Polish Academy of Sciences, Wrocław, Poland; ^5^ Department of Pharmacology, Oslo University Hospital, Oslo, Norway; ^6^ Department of Internal Medicine, Division of Hematology, Medical University of Graz, Graz, Austria

**Keywords:** chronic graft-versus-host disease, hematopoietic stem cell transplantation, immune cell networks, cell-cell communication, GvHD pathogenesis

## Abstract

Chronic Graft-versus-Host Disease is a life-threatening inflammatory condition that affects many patients after allogeneic hematopoietic stem cell transplantation. Although we have made substantial progress in understanding disease pathogenesis and the role of specific immune cell subsets, treatment options are still limited. To date, we lack a global understanding of the interplay between the different cellular players involved, in the affected tissues and at different stages of disease development and progression. In this review we summarize our current knowledge on pathogenic and protective mechanisms elicited by the major involved immune subsets, being T cells, B cells, NK cells and antigen presenting cells, as well as the microbiome, with a special focus on intercellular communication of these cell types *via* extracellular vesicles as up-and-coming fields in chronic Graft-versus-Host Disease research. Lastly, we discuss the importance of understanding systemic and local aberrant cell communication during disease for defining better biomarkers and therapeutic targets, eventually enabling the design of personalized treatment schemes.

## Introduction

For patients with hematologic malignancies, primary immunodeficiencies or bone marrow (BM) failure, allogeneic hematopoietic stem cell transfer (HSCT) is often the only option for curative treatment. This treatment, however, comes with a high risk of developing acute or chronic Graft-versus-Host Disease (GVHD). While acute GVHD (aGVHD) occurs early after transplantation and mainly affects the skin, liver and gut, chronic GVHD (cGVHD) establishes later after transplantation and is a heterogenous syndrome with multi-organ involvement. Patients typically require long-term treatment with immunosuppressive drugs and are faced with a high risk of mortality and severe impact on their quality of life. The pathogenesis of cGVHD involves multiple processes, which are complex and inter-dependent. Recently, a 3-phase model for cGVHD was proposed that involves early inflammation and tissue injury including thymic damage with loss of central and peripheral tolerance, subsequent chronic inflammation and dysregulated immunity and eventually aberrant tissue repair with fibrosis (1).

### A diverse set of immune cellular players mediate cGVHD

Every phase of cGVHD involves a diverse set of immune cells, which exert several disease-promoting pathomechanisms. The precise interplay between these cell types and mechanisms on a systemic level as well as in the affected tissues is incompletely understood. In this review we give an overview of the different immune components involved in cGVHD pathogenesis and summarize our current knowledge about dysregulated cell composition, signaling and intercellular communication in cGVHD. Specifically, we address mechanisms and therapeutic potential of T cell regulated pathogenic signaling and give an overview of disturbances in the B cell compartment. We discuss the dual role of NK cells, touch upon the role of mixed chimerism of antigen presenting cells (APCs), macrophage polarization and structural cells as APC in disease pathogenesis, and outline immunomodulatory mechanisms mediated by the gastrointestinal microbiome in cGVHD.

### Cellular crosstalk in cGVHD *via* extracellular vesicles – a new field of GVHD research

The different cell types involved in disease pathogenesis do not act as single entities but are communicating with each other to facilitate their effector functions. Extracellular vesicles (EVs) play a central role in immune cell crosstalk and represent an entirely new area for research in the context of GVHD.

All eukaryotic cells release EVs that contain a subset of proteins, lipids and nucleic acids, predominantly small RNA (smRNA), that are derived from the parent cell. These particles vary in generation method and size, including shedding microvesicles (1000-100nm) and exosomes (150-30nm) from multivesiculated bodies generated in the endocytic pathway. Therefore, EVs are heterogeneous and differ in their content and surface proteins. It is now well established that EVs play an important role in intercellular communication by transferring their contents between cells (2). They are also involved in numerous essential physiological processes, and vesicles from both non-immune and immune cells have important roles in immune regulation (3). EVs play a role in both innate (4) and adaptive immunity where they can have both pro- and anti-inflammatory capabilities, depending on their contents. They can serve as disease biomarkers for aGVHD, as recently shown by Lia et al (5), but are likely involved in several GVHD promoting mechanisms by facilitating cell-cell communication. EVs in GVHD have so far mostly been studied in the context of mesenchymal stem cell (MSC)-based therapy. Several mouse models and few studies in human subjects revealed that the immunomodulatory properties of MSC are largely mediated by their secretome, which includes EVs. These effects have recently been extensively reviewed in Doglio et al (6) and Lia et al (7). However, also immune cells themselves are releasing EVs that have the potential to exert autocrine and paracrine immunomodulatory effects. These mechanisms are this far understudied in GVHD. In the following chapters we summarize recent findings regarding EVs produced by a variety of immune cells which are known to be involved in the etiology of GVHD, discuss their effects and how they could shape the inflammatory environment during active GVHD.

The role of EVs in the pathophysiology of immunological disorders is an up-and-coming field for research, as they offer translational importance as prospective therapeutic targets, informative biological agents, and predictive disease biomarkers.

## Novel mechanisms of T cell pathogenicity in cGVHD

**Figure 1:**
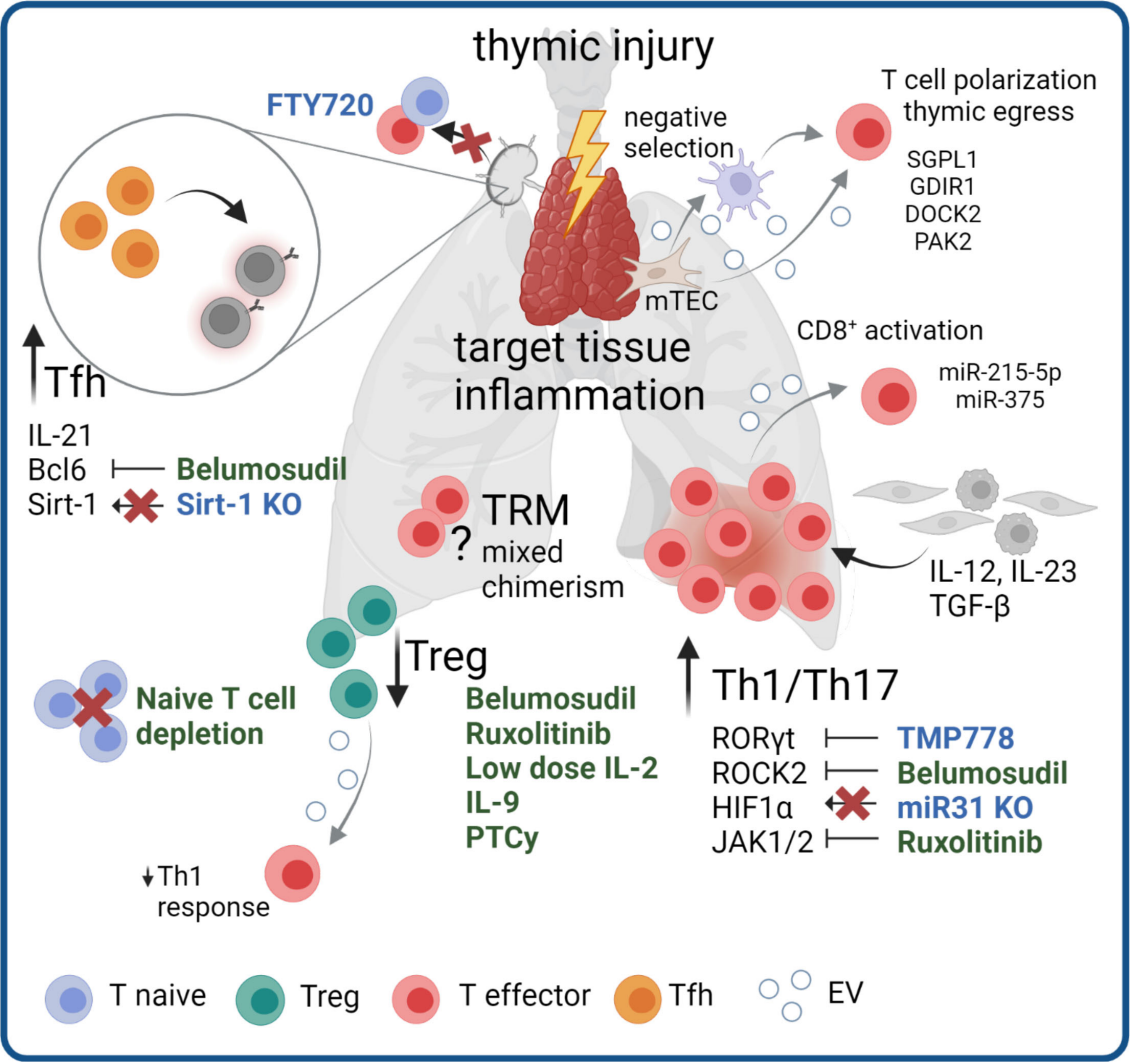
Mechanisms and targeting of T cell pathogenicity in cGVHD. Pathogenic mechanisms in cGVHD include thymic injury and subsequent dysregulation of Treg responses and Th1/Th17 skewing. Pro-inflammatory signaling from other cell types promotes inflammation in target tissues. Increased numbers and signaling of Tfh additionally stimulate pathogenic B cell responses. The pathogenic role of mixed chimerism within TRM compartment and potential antigens for a specific allo-reaction remain topics of open investigation. Therapeutic agents in clinical use (green) or agents to reduce cGVHD in pre-clinical models (blue) targeting the different disease mechanisms are highlighted. Grey arrows indicate potential involvement of EV in pathological processes.

The pathophysiology of cGVHD is complex and involves a plethora of different organ systems and cell types, and both innate and adaptive immune responses are involved. Especially in the second phase of cGVHD development with chronic inflammation and dysregulated immunity, adaptive immune responses are initiated and result in a loss of central and peripheral tolerance and the emergence of pathogenic T cells and B cells, causing auto- and alloimmune reactions. In this chapter we highlight novel players identified in pathogenic T cell signaling in cGVHD and how communication *via* EVs could contribute to pathogenic effects (**Figure 1**).

### Th1/Th17 polarized T cells dominate inflammatory responses in cGVHD

Different subsets of CD4^+^ T cells have been reported to have crucial roles in disease development and maintenance. Principally, cGVHD is dominated by Th1/Th17-like immune responses, with a simultaneous loss of Treg function. Thymic injury during disease progression leads to diminished Treg generation and release of autoreactive T cells becoming IL-17A producing effectors (reviewed in (8)). Recently, Alawam et al. reported a failed recovery of medullary thymic epithelial cells as the main mechanism for impaired Treg development and escape of self-reactive conventional αβ T cells in mice after syngeneic BM transplantation (9). T cells with Th1/Th17 polarization are enriched in cGVHD target tissues such as the skin and liver (10, 11), and are thought to mediate chronic inflammation. The Th1/Th17 skewing in cGVHD target tissues is driven by mediators produced by other immune and stromal cells within these tissues. Thereby, the IL-12 cytokine family plays an important role, as IL-12 and IL-23 are the major drivers of Th1 polarization and Th17 maintenance (reviewed in (12)). The main cytokine sources are activated macrophages and dendritic cells in tissues, although IL-23 production by keratinocytes has been described in the context of skin inflammation (13, 14). Keratinocytes and other epithelial cells produce TGF-β, an important cytokine for Th17 as well as Treg polarization, wherein the former additionally requires the presence of IL-6 or IL-21 (15). IL-6 can be produced by many stromal and immune cell types, whereas IL-21 production is restricted to activated T cells.

Recently, Forcade et al. showed that cGVHD patients represent with elevated frequencies of CD146+ CCR5+ T cells, which have enhanced migration potential and Th1/Th17 features (16). The same study could show that TMP778, a small molecule inhibiting Th17 master transcription factor RORγt, alleviated murine cGVHD (16). Approaches to target Th1/Th17 cells to prevent or revert cGVHD have become a topic of increasing interest. A recent mouse study revealed a role for microRNA (miR) -31 in T cell pathogenicity in cGVHD. miR-31 deficient T cells displayed reduced Th17 differentiation and increased Treg generation through loss of HIF1α upregulation in allogeneic cells, leading to improved cutaneous and pulmonary cGVHD (17). Of note, HIF1α signaling is known to boost Th17 skewing in T cells and can be induced by TCR signaling upon target cells recognition, cytokine signaling, or hypoxia, which is a hallmark of inflamed tissues (reviewed in (18)). Pre-clinical findings show that ROCK2 inhibition reduces production of Th17 signature cytokines IL-17 and IL-21 in a STAT3-dependent manner, while increasing STAT5 signaling, thereby promoting Treg function (19, 20), have led to clinical studies testing ROCK2 inhibitors in GVHD, which we discuss later in this chapter.

### Dysregulated regulatory T cells prompt failed tolerance

Dysregulated Treg immunity is another hallmark feature of cGVHD (21, 22). Failure to properly reconstitute the Treg compartment after HSCT results in loss of tolerance, allo- and autoimmunity and impaired control of immune responses (21, 22). Importantly, Treg expand in secondary lymphoid organs and subsequently migrate to peripheral tissues, where they suppress local inflammatory responses and prevent the early expansion of alloreactive effector cells (23, 24). Low-dose IL-2 therapy for Treg induction has shown beneficial effects on Treg numbers in cGVHD, albeit a considerable number of patients are unresponsive (25–27). A recent study demonstrated preferential activation of effector T cells over Tregs in a severely inflammatory environment and therefore suggested IL-2 treatment to be tailored to the immune status of each patient (28). Nicholls et al. investigated BM residing Tregs (BM-Treg) in the context of cGVHD, which failed to reconstitute properly and did not expand in response to IL-2 but rather IL-9. Importantly, BM-Treg depletion, while maintaining splenic Treg, resulted in exacerbated skin cGVHD (29). Type 1 adaptive regulatory cells (Tr1) are another type of Treg, characterized by absence of Foxp3 expression, high IL-10 production, and co-expression of CD49b and LAG-3 (30), and are crucial to control aGVHD in mice (31). In human cGVHD, the Tr1 subset was shown to be specifically enriched in patients with active disease compared to patients in remission, whereas the inverse was true for thymic Treg (32). However, the functional role of Tr1 in disease control needs to be further addressed. Over the past years post-transplant cyclophosphamide (PTCy) has emerged as a promising and effective treatment strategy to reduce the incidence of cGVHD. Although initially the effect was attributed to a general impairment of allo-reactive donor T cells, newer studies found a Treg-specific sparing effect (33–35). Comparing T cells in patient blood receiving PTCy with no PTCy regimens revealed a significantly delayed T cell reconstitution with a simultaneous increase in the proportion of Treg in the PTCy group (35). PTCy patients also displayed a higher level of inhibitory markers on both CD4^+^ and CD8^+^ cells, with reduced capacity for cytokine production after ex vivo stimulation, giving new insights into the mechanism of action of PTCy mediated cGVHD prevention.

### Follicular helper T cells enable B cell pathogenicity

Another T cell subset critically implicated in cGVHD pathogenesis are follicular helper T cells (Tfh), a subset of CD4^+^ T cells located in the B cell follicle and expressing the transcription factor Bcl6 along with high levels of the chemokine receptor CXCR5 and programmed cell death protein-1 (PD-1), Tfh cells are crucial for initiating B cell-mediated disease mechanisms by enabling germinal center formation through IL-21 production, and increased Tfh numbers correlate with disease severity (36). ROCK2 inhibition in murine cGVHD, in addition to regulating the Th17/Treg balance, decreased the Tfh master transcription factor Bcl6 and consequently the frequency of Tfh (19). Another mouse study proposes Sirt-1 as a new cGVHD target, as T cell-specific Sirt-1 deficiency negatively affected Tfh generation, B cell activation and plasma cell differentiation, while simultaneously promoting Treg stability (37). Similarly, IL-39, a recently described IL-12 family cytokine, aggravated cGVHD in mice by promoting STAT1/STAT3 mediated CD4^+^ T cell activation and differentiation of germinal center B cells in mice after allogeneic (allo-)HSCT (38). This study described activated B cells, CD11b^+^ cells and CD8^+^ T cells as main IL-39 sources. However, there is so far no evidence for a pathogenic function of IL-39 in human GVHD.

### T cell targeting strategies are promising new therapeutic

The overall importance of dysregulated T cell activation and polarization of T cell subsets in cGVHD pathogenesis is underlined by the therapeutic efficacy of Ruxolitinib, a selective Janus kinase (JAK) 1/2 inhibitor. Blocking JAK/STAT signaling affects different aspects of T cell biology related to cGVHD pathogenesis, such as production of pro-inflammatory cytokines, proliferation and survival and the differentiation into IFN-γ and IL-17A producing cells (39). In September 2021, the FDA approved Ruxolitinib for cGVHD after failure of one or two lines of systemic therapy in adults and children >12 years, based on results of the REACH-3 (NCT03112603) trial (40). Additionally, the selective JAK1 inhibitor Itacitinib in combination with corticosteroids is currently tested as first line therapy for cGVHD (NCT03584516). As mentioned above, promising results for ROCK2 inhibition were obtained in pre-clinical models. Belumosudil, a selective ROCK2 antagonist, was tested for safety, tolerability, and activity in cGVHD patients in clinical studies (NCT02841995, NCT0344081) (41, 42). These studies included heavily pre-treated refractory patients having failed one or more previous lines of therapy and reported overall response rates (ORR) of > 70% (41) and > 60% (42), respectively, in all treatment groups, with limited cytotoxicity and improved quality-of-life. These results suggest Belumosudil therapy to be suitable for patients with treatment-refractory cGVHD, which resulted in FDA approval for cGVHD patients 12 years or older after failure of two or more prior systemic treatments in July 2021 (43).

Additional attempts to prevent the dissemination of allo- and autoreactive T cells include the depletion of naïve T cells before transplantation and blocking of T cell migration. Since mouse studies demonstrated exacerbated disease mediated by naïve T cells compared to memory T cells (44, 45), and naïve T cells numbers correlate with the later onset of cGVHD and are elevated in starting cGVHD in patients (46), clinical studies on the efficacy of naïve T cell depletion from the graft were conducted. Recently published results from three clinical trials (NCT00914940, NCT01858740, NCT02220985) revealed a very low rate of cGVHD in those patients (47). From 138 patients, 1% developed moderate-severe cGVHD and the 3-year GVHD-free, relapse-free survival was 68%, suggesting naïve T cell depletion could be a novel superior strategy of graft engineering (47). In mice, application of the S1P agonist FTY720, which blocks lymphocyte migration and traps naïve and effector T cells in lymph nodes, reduced cGVHD severity and fibrosis (48). Of note, according to a case report by Gauthier et. al, a patient with severe central nervous system GVHD was successfully treated with FTY720 (49).

### Tissue T cell chimerism can drive inflammation

So far, efforts to uncover T cell pathogenicity in GVHD have focused on donor T cells. However, it has lately become evident that host-derived tissue-resident memory T cells (TRM) can survive the conditioning regimen and persist in GVHD target tissues, such as the skin and the gut (50–52). The presence of host TRM in other GVHD target tissues has this far not been reported but is likely due to the high abundance of TRM in tissues such as liver and lung (53, 54). In patients’ skin with active GVHD, these host-derived T cells were proliferative and produced effector cytokines (51, 52). Data obtained in a cutaneous GVHD humanized mouse model demonstrated that host TRM activated by donor APC are sufficient to induce a GVHD-like dermatitis (51). Host T cells with a resident phenotype could be found in increased numbers in the blood of aGVHD patients and were able to mediate keratinocyte damage in an *ex vivo* model (55). On the other hand, in murine GVHD models, mice receiving T cell depleted bone marrow usually don’t develop skin manifestation. This discrepancy may result from species differences in tissue resident T cell biology, and artifacts such as low abundance of antigen experienced TRM in laboratory mice compared to humans or pet store “dirty” mice (56). In fact, patients receiving T cell depleted PBSCT can still develop cutaneous aGVHD (57), underlining the importance to study disease mechanisms in human models to translate pre-clinical findings.

Host TRM in the skin were reported to have low replacement rates and persist in the skin at ranges of 5-95% 10 years after transplantation (52). Another study reports that 23% of patients display long-term skin T cell chimerism (50). Interestingly, in this study skin T cell chimerism occurred in 57% of patients with aGVHD, but only 12.5% of patients with cGVHD. Importantly, these studies demonstrated that a previous history of GVHD correlated with a loss of host TRM, indicating that host TRM might be preferentially targeted by infiltrating alloreactive donor T cells (50, 51). These data suggest that host TRM, while being dispensable for GVHD development, can contribute to GVHD related inflammation. However, the specific pathogenic mechanisms, and especially a potential role of host TRM in cGVHD pathology, must be further investigated. In the future, the development and use of TRM specific depletion models for specific organs should give clearer insights into the role of these cells in aGVHD and cGVHD.

### EVs mediate T cell pathogenic signaling

EVs play a role in the development of T cells. Thymic epithelial cells have been shown to release EVs containing tissue restricted antigens (58) which are then presented to thymic cDCs for antigen presentation, contributing to the negative selection of T cells with specificity to self-antigens (58). In the context of cGVHD, this may have an important effect on the maintenance of central tolerance, due to potential changes in the type and content of EVs during thymic damage. More specifically, EVs containing molecules such as sphingosine-1-phosphatase lyase 1 (SGPL1), Rho GDP-dissociation inhibitor 1 (GDIR1), dedicator of cytokinesis protein 2 (DOCK2), and p21 protein-activated kinase 2 (PAK2) contribute to the polarization of CD4 or CD8 single positive thymocytes, aiding both maturation of T cells and thymic egress, but continue to contribute to T cell function in the periphery (59).

The ability of Tregs to enforce peripheral tolerance, and suppress Th1 cell responses, has been shown to be partly *via* nonautonomous gene silencing, mediated by miRNA-containing EVs (60). Indeed, Tregs are prolific producers of EVs that contain high levels of miRNA, the profile of which is distinct from those of Th1 and Th2 cells. Treg derived EVs can transfer specific sets of miRNA to conventional T cells, both *in vitro* and *in vivo* (60, 61). Compromised transfer of Treg EV miRNAs to conventional T cells (either *via* failed miRNA formation (Treg cell Dicer deficiency), or inhibited EV release (Rab27a- and Rab27b-deficient Tregs) has been shown to abrogate the capacity of Tregs to prevent disease in a colitis model, by specifically regulating Th1 cell responses (60). The specific role of Treg EV mRNA and proteins in modulating target cells in a context-dependent manner remains unknown, but seems likely to drive their immunomodulatory effects. Loss of Tregs and their tolerogenic function enables pro-inflammatory immune cells to perpetuate GVHD.

Overall, T cells remain the most studied immune cell subset in both aGVHD and cGVHD, as they are crucial for disease development and maintenance and the most obvious target for preventive and curative therapies. However, many studies focus on the blood, studying systemic disease effects. With our improved understanding of tissue immunity and the diverse phenotypes T cells can acquire in different organs, there is a need to investigate tissue-specific disease mechanisms. A recent study in a murine GVHD model demonstrated that disease is maintained in target organs solely by resident T cells and their progenitors (62), highlighting the need to develop tissue-targeting therapies.

## Communication of immune cells in cGVHD – the role of B cells

**Figure 2:**
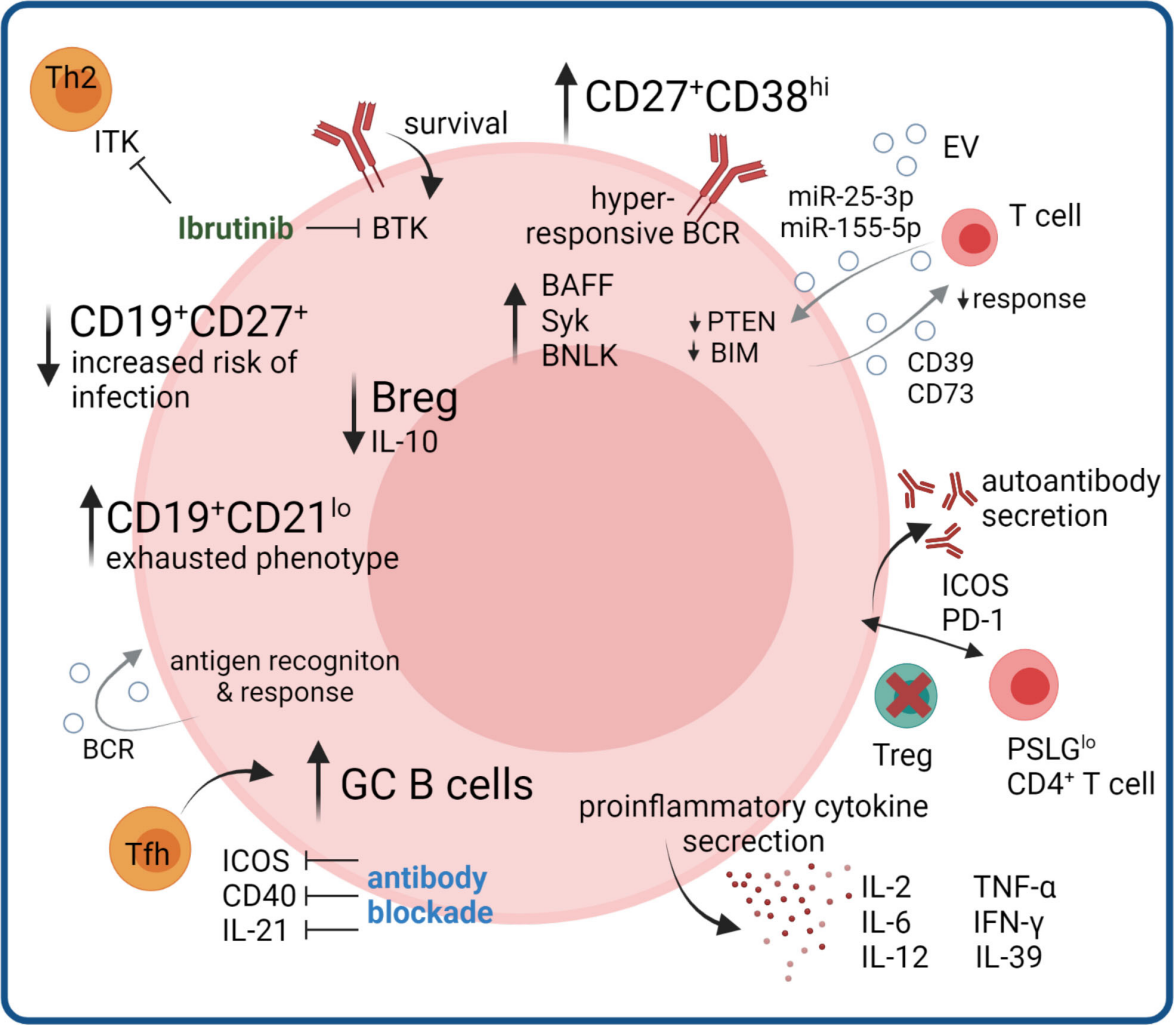
B cell compartment aberrations in cGVHD. B cell compartment aberrations include increases in memory B cells with exhausted phenotype, and in germinal center GC B cells promoted by increased Tfh signaling, where therapeutic benefit could be seen by monoclonal antibody blockade in mouse models. Hyperresponsive BCR signaling and autoantibody production, as well as production of pro-inflammatory cytokines further promotes disease pathogenesis, partly mediated by loss of Treg-controlled B cell help. Regulatory B cell functions are diminished, as well as certain memory subsets controlling infections. Ibrutinib inhibits the B cell survival regulating kinase BTK and additionally suppresses Th2 responses via blocking ITK. B cell derived EV signals regulating T cell and B cell activation could influence proinflammatory immune responses.

The lymphoid system is known to be a target organ of cGVHD seen in lymphoid hypocellularity, functional asplenia and humoral immune deficiency (63–65). B cells have a substantiated role in cGVHD including dysregulation of the B cell compartment, aberrant B cell signaling pathways, production of allo- and autoantibodies and T – B cell interactions. Aberrant B cell homeostasis with reduced generation of BM B lymphoid progenitors (65–68), low frequencies of naïve and memory B cells (69, 70) and a regulatory B cell defect have been observed in cGVHD (**Figure 2**) (71). Here, we will give an overview of aberrant B cell subpopulations and B cell signaling in cGVHD and highlight abnormalities in T and B cell crosstalk.

### Aberrant B cell subpopulations and signaling pathways characterize cGVHD

Significantly higher relative numbers of CD19^+^CD21^low^ B cells in patients with long-lasting or first diagnosis of cGVHD were reported and this B cell subpopulation significantly correlated with activity and severity of cGVHD (46, 69, 70, 72–75), respectively. In addition, CD19^+^CD21^low^ B cells were elevated on day +100 after allo-HSCT in patients subsequently developing cGVHD (46). Khoder et al. further characterized the CD19^+^CD21^low^ B cell subset demonstrating features of exhaustion, such as increased expression of multiple inhibitory receptors, altered expression of chemokine and adhesion molecules and poor proliferative response to a variety of stimuli (72). Further research with modern single-cell approaches combining antigen specificity and phenotype will elucidate whether the exhaustion results from constant antigen exposure or from of unspecific activation. It is also unclear whether exhausted B cells affect cGVHD pathogenesis, are a mere consequence of persistent inflammation or a cause of defective B cell reconstitution. A recent single cell study reported the expansion of CD27^+^ memory and CD11c^+^ atypical memory B cells in cGVHD patients, which can exhibit an exhausted phenotype. Trajectory analysis predicted that the cGVHD-expanded cluster develops along different trajectories compared to corresponding healthy control populations, bypassing tolerance checkpoints (76).

Both B cell receptor (BCR) signaling and B cell activation factor (BAFF) play key roles in determining B cell fate and survival. Multiple groups have shown that cGVHD is closely associated with aberrant BAFF levels (46, 67, 74, 77, 78), an activated B cell phenotype, and aberrant BAFF/B cell ratios (46, 67, 74, 78, 79). Excessive BAFF levels have been associated with increased proportions of CD27^+^ B cells, including both pre-germinal center (GC) CD27^+^CD38^hi^IgD^+^ and post-GC CD27^+^CD38^hi^IgD^-^ B cells in patients with active cGVHD as compared to healthy individuals (78). B cells from cGVHD patients had significantly increased proliferative responses to B cell receptor (BCR) stimulation and resistance to apoptosis, resulting in the constant activation of B cells (80–82). BCR hyperresponsiveness is reportedly associated with increased expression of proximal signaling molecules including spleen tyrosine kinase (Syk) and B cell linker (BLNK) protein (81), an effect known to be mediated *via* increased BAFF levels. Activated CD27^+^ B cells from cGVHD patients are in an increased metabolic state with bigger size and protein content and are primed for survival *via* BAFF pathways (80). The Syk inhibitor Fostamatinib was recently tested in a Phase I clinical trial (NCT02611063) for cGVHD prophylaxis and as steroid-refactory cGVHD treatment (83). In both arms the proportion of post-GC plasmablas like B cells decreased in the early treatment phase (83). Bruton’s tyrosine kinase (BTK) is downstream of the BCR and required for tonic and antigen-dependent receptor signaling which regulates B cell survival (84). Ibrutinib, an FDA-approved drug for steroid-refractory cGVHD inhibits BTK in B cells, leading to reduced production of autoreactive antibodies but also inhibits a homologous enzyme in T cells, interleukin-2-inducible T cell kinase (ITK), leading to selective suppression of Th2 immune responses that may contribute to the pathogenesis of cGVHD (85, 86). However, recent results from the iNTEGRATE study (NCT02959944), evaulating first-line treatment of cGVHD with iburitinib-prednisone showed no benefit compared to placebo-prednisone treatment, which may be attributed to different patient cohorts and study desing or reflect different immune mechanisms active in steroid-refractory compared to untreated cGVHD (87). In mice with cGVHD combinatorial treatment with BTK and ITK was beneficial (88), warranting further studies to explore the benefit of combination therapy in humans.

Another hallmark of cGVHD is the lower frequency of CD19^+^CD27^+^ memory B cells (69, 70), whose reduced number is associated with the incidence of infectious complications after allo-HSCT (69, 70, 74). Of note, resolution of cGVHD correlated with expansion of CD19^+^CD27^+^ memory B cells (69). Using B cell rearrangement excision circle measurements, Glauzy et al. observed increased B cell replication but decreased overall B cell neogenesis in patients with aGVHD and cGVHD (67).

B cells can have regulatory properties that suppress rather than initiate cGVHD, and regulatory B (Breg) cells have been identified as a novel B cell subpopulation associated with cGVHD development (71, 89, 90). Compared to healthy individuals, cGVHD patients have decreased Breg cells defined as interleukin (IL)-10 secreting cells with reportedly different phenotypes. Khoder et al. defined B cells with immunoregulatory properties within both the CD19^+^IgM^+^CD27^+^ memory and CD19^+^CD24^high^CD38^high^ transitional B cell compartments and observed a lower frequency of Breg cells in patients with cGVHD (89). Furthermore, Breg cells in these patients were less likely to produce IL-10 compared to Bregs from healthy donors. De Masson et al. observed enriched IL-10 production in both the CD24^hi^CD27^+^ and CD27^hi^CD38^hi^ plasmablast B cell compartments (71). Patients with cGVHD had less CD24^hi^CD27^+^ B cells and IL-10–producing CD24^hi^CD27^+^ B cells and increased CD27^hi^CD38^hi^ plasmablast frequencies, but decreased IL-10–producing plasmablasts (71).

### Abnormal T and B cell interactions characterize cGVHD

Antigen presentation by activated B cells that have up-regulated major histocompatibility complex (MHC) and costimulatory molecules, such as CD80 and CD86, leads to CD4^+^ and CD8^+^ T cell activation and differentiation. Depending on the B cell subpopulation and the nature of the activation stimulus, B cells can also act as immunoregulatory cells that induce peripheral CD4^+^ and CD8^+^ T cell tolerance, inhibit dendritic cells, and induce and expand regulatory T (Treg) cells.

Amongst other mechanisms, cGVHD is mediated by abnormal CD4^+^ T and B cell interactions, due to the lack of donor-derived Treg cells (91, 92). B cells contribute to immune responses by antibody-mediated and antibody-independent mechanisms. Antibodies directed against Y-chromosome-encoded epitopes (H-Y antibodies) in male recipients of stem cell grafts from female donors developing cGVHD have been observed (93–95). The presence of auto-antibody-secreting B cells promoted by alloreactive donor CD4^+^ T cells is an important mediator of autoimmune features of cGVHD, suggesting that a loss of B cell tolerance is operative (92, 96, 97). A number of autoantibodies, including anti-nuclear antibodies (ANAs), anti–double-stranded DNA (anti-dsDNA), and anti-platelet-derived growth factor receptor (PDGFR)-α, have been found in association with cGVHD (98–102).

In a preclinical cGVHD model, B cells were required to induce cGVHD and associated bronchiolitis obliterans syndrome (BOS) (103). Of note, tissue fibrosis was observed only after secretion of high-affinity class-switched antibodies that activate immune responses and, in the case of cGVHD, cause severe damage to target tissues by activating complement or antibody-dependent cell-mediated cytotoxicity. Furthermore, in this well-established murine cGVHD model, donor T cell–derived Tfh cells were required for the activation of donor BM–derived B cells that differentiate into GC B cells, resulting in immunoglobulin deposition in BOS lesional tissue (104). Increased frequency of Tfh cells correlated with increased GC B cells, cGVHD, and BOS. Development of BOS was dependent upon T cells expressing the chemokine receptor CXCR5, to facilitate T cell trafficking to secondary lymphoid organ follicles. Tfh cells support the generation of GCs by providing signaling through IL-21, inducible T-cell co-stimulator (ICOS), and CD40 (105–108); blocking these factors by monoclonal antibodies hindered GC formation and cGVHD (104).

In a murine model, Deng et al. observed that cGVHD causes destruction of lymphoid follicles and absence of GCs in lymphoid tissues (109). Development of cGVHD did not require GC formation and was associated with the expansion of extrafollicular CXCR5^-^BCL6^+^PSGL1^low^CD4^+^ T cells in GVHD target tissues (109). The same research group demonstrated that murine and human PSGL1^low^CD4^+^ T cells from GVHD target tissues have features of B cell helpers with upregulated expression of PD1 and ICOS and production of IL-21 (110). Murine PSGL1^low^CD4^+^ T cells from GVHD target tissues augmented B cell differentiation into plasma cells and production of autoantibodies *via* their PD1 interaction with PD-L2 on B cells. As mentioned before, the use of PTCy is increasingly applied to prevent cGVHD and a recent study in murine cGVHD suggests a critical role for Treg-B cell interactions in its effect. In this model PTCy, but not other prophylactic regimens inhibited initial alloreactive T cell responses, which led to increased Tregs and restored bone marrow B cell lymphogenesis (111).

Additional pathologic functions of B cells such as production of a large number of proinflammatory cytokines including IL-2, tumor necrosis factor (TNF)-α, IL-6, IL-12, and interferon-γ that activate immune cells such as T cells, macrophages, and natural killer (NK) cells have been shown to be involved in cGVHD pathogenesis (1, 8). Recently, Lv et al. observed that amongst other cells also activated B cells can produce IL-39, which might promote the expression of IL-39 receptors on CD4^+^ T cells (38). Overexpression of IL-39 promoted the aggravation of cGVHD development in two different murine models, suggesting a critical role of IL-39 in the pathogenesis of cGVHD (see previous chapter).

In conclusion, B cell subpopulations and their associated signaling pathways as well as coordinated T cell and B cell responses have a key role in the pathogenesis of cGVHD. Pharmaceutical attempts to restore B cell homeostasis and target rare, pathogenic B cell subpopulations offer a new opportunity to reverse dysregulation of adaptive immunity in cGVHD.

### B cell-derived EVs regulate T cell activation

When stimulated with CD24 or IgM, B cells are able to transfer functional BCR to recipient B cells *via* EVs (112). This was demonstrated by Phan et al. in a murine knockout model, where donor cells expressing GFP were co cultured after stimulation with IgM and CD24 knockout cells, resulting in the transfer of lipids and functional BCR to recipient cells, allowing for recognition of antigen and response (112). EVs from ‘healthy’ B cells are an area of limited research, however, there are interesting findings from B cell malignancies. Chronic lymphoid leukemia (CLL) B cells stimulated with CD40 and IL-4 release EVs containing miR-363, which can be transferred to CD4^+^ T cells and has a repressive effect on expression of CD69, a marker of activation in T cells (113). In this way, not only can more prominent immunosuppressive cell types such as Tregs or DCs subdue effector cells, but B cells can also repress CD4^+^ T cells. Co-stimulatory CD40L from EVs has also been demonstrated to have an effect on B cell response (114), whereby CD4^+^ T cell derived EVs that contain CD40L in their cargo, rather than on their surface, aid in the efficacy of HBsAg vaccination, increasing B cell activation, proliferation, and antibody production without impacting the surface expression of CD40 or CD80 on B cells (115). While this type of response is beneficial in the context of vaccination, increased aberrant B cells and concomitant antibody production could lead to aggravation of cGVHD. EV transfer of microRNAs from T cells to B cells also affect the immune response. Indeed, EV transfer of miR-25-3p and miR-155-5p, alongside other miRNAs such as miR-20a-5p, resulted in the silencing of the phosphatase and tensin homologue (PTEN) and Bcl-2-interacting mediator of cell death (BIM) genes. Both are known to have a critical function in B cell biology and the germinal center reaction, suggesting their role in germinal center formation and antibody production (116). Therefore, future research is needed to determine if aberrant EV signaling from B cells are amplifying disease mechanisms in the context of cGVHD.

Substantial dysregulation in the B cell compartment, in subset composition, pro-inflammatory signaling and disturbed T cell crosstalk is a specific feature of cGVHD and not aGVHD and therefore is critically important to understand in order to prevent and effectively treat this specific disease manifestation after HSCT. Restoring T cell function impacts the B cell compartment and vice versa, however, multiple factors determine these interactions and although there are many promising therapies recently approved or in the pipeline, it is still hard to predict which patients respond best to which treatment. Investing research in robust biomarkers for both B and T cell dysregulation as the two major adaptive immune arms driving pathology will help to stratify patients and improve treatment plans.

## NK cells communicating within the cGVHD microenvironment

**Figure 3:**
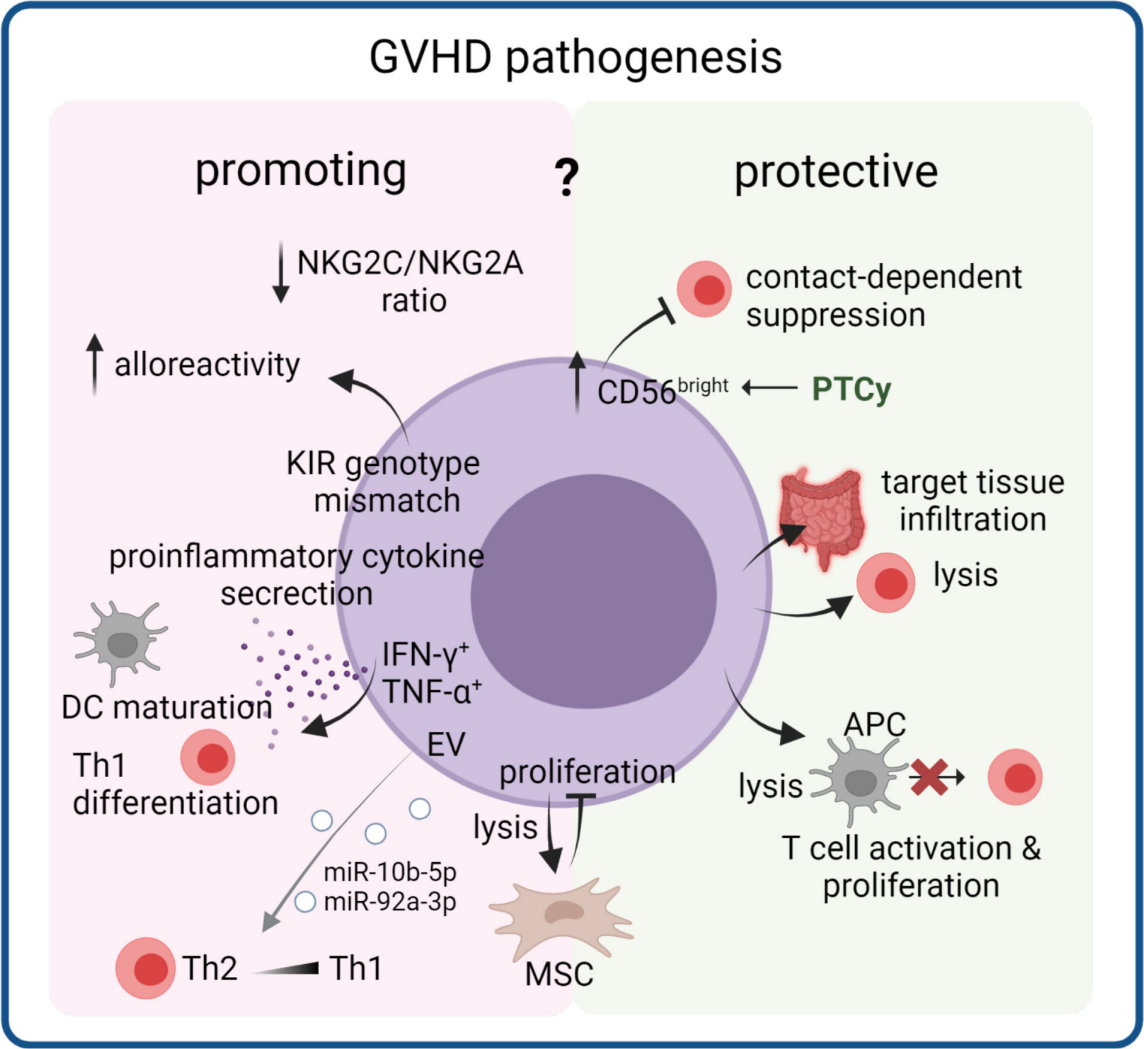
Dual role of NK cells in cGVHD pathogenesis. NK cells that reconstitute early after transplantation can acquire several properties that render them GVHD promoting or GVHD protective. Through lysis or suppression of T cells or APC in the circulation and in target tissues they can reduce inflammation. On the other hand, proinflammatory cytokine production and increased alloreactivity through a donor NK cell receptor (including KIR) repertoire can support disease pathology. Their interaction with mesenchymal stem cells (MSC) could modulate effects of MSC-based therapies. EV released from NK cells can induce Th1 polarization, thereby potentially contributing to cGVHD pathology. .

Natural killer (NK) cells are the first reconstituted immune cells to appear after allo-HSCT (117). Additionally, donor-derived NK cell infusion is increasingly studied as a prophylactic measure after allo-HSCT to target residual cancer cells (118, 119). However, the role of NK cells in cGVHD is not clear (120), and may depend on various factors and interactions within the microenvironment (**Figure 3**).

### Activating and inhibitory NK cell receptors display dynamic expression patterns after HSCT and in cGVHD

Specific activity of NK cells is fine-tuned by expression of both activating and inhibitory receptors, which form a system of dynamic equilibrium that regulates NK cell responses (121). Among the various receptors present on NK cells, killer immunoglobulin-like receptors (KIR) (122) are a family of highly polymorphic receptors that includes members with both activating and inhibitory properties (123, 124). KIRs mostly recognize HLA class I molecules as their ligands (125, 126). KIR genotype mismatching was shown to increase cGVHD in HSCT patients undergoing myeloablative conditioning (127). Furthermore, KIR licensing status was significantly associated with cGVHD incidence in a univariate analysis (128).

Aside from KIRs, there is a wide variety of other NK cell receptors of both activating and inhibitory function. While not as polymorphic as KIRs, the genes coding for these receptors and their ligands harbor various important single nucleotide polymorphisms (SNPs) that can affect their function or expression (129), and may be associated with GVHD, including its chronic form (130). The natural cytotoxicity receptor (NCR) family includes three activating NK cell receptors: NKp46 (NCR1), NKp44 (NCR2), and NKp30 (NCR3) (131–133) with ligands that are not well defined (134–138). Another important NK cell receptor is DNAX Accessory Molecule-1 (DNAM-1), also known as CD226 (139). Its ligands are the adhesion proteins CD112 and CD155 (140). The CD92/NKG2 family includes both activating (e.g. NKG2C) and inhibitory receptors (e.g. NKG2A), encoded by genes located on chromosome 12 (141). The activating/inhibitory pair NKG2C/NKG2A is particularly important in NK cell maturation after HSCT. Interestingly, both receptors were shown to be downregulated in patients with extensive cGVHD (as compared to patients with no or mild cGVHD). However, the NKG2C/NKG2A ratio was decreased, suggesting a less activating profile of NK cells in patients developing extensive cGVHD (142).

### NK cells communicate with other cGVHD cellular players

NK cells use their receptors and cytokine release to interact with other cells. They can stimulate maturation of APCs such as dendritic cells (DCs) by releasing TNF-α and IFN-γ and can also kill immature DCs *via* NKp30 and DNAM-1 receptors (143–145). Cytokine secretion by DCs themselves can promote cytotoxicity and cytokine production of NK cells (146). In mice, IFN-γ secretion by NK cells was able to promote differentiation from naïve CD4^+^ T cells to Th1 cells, which are important in cGVHD development (147–149). In both mice and humans, activated NK cells can specifically kill activated and proliferating CD4^+^ and CD8^+^ T cells in a perforin-dependent manner. This process is mediated by NK cell receptors NKG2D and DNAM-1, whose expression may be upregulated on activated T cells (150–152). NK cells can also suppress CD8^+^ T cells by releasing IL-10 (153, 154).

Mesenchymal stem cells (MSCs) are another cellular subpopulation known to interact with NK cells. A study on IL-2-activated NK cells showed that they can lyse MSCs, and this action likely involved NKp30, NKG2D, and DNAM-1 receptors. At the same time, MSCs inhibited proliferation of resting NK cells (155). The decreased NK cell proliferation corresponded with increased cytokine secretion and cytotoxicity, although some studies found these NK cell functions to be decreased instead (156–159). NK cell interactions with MSCs are of particular interest, due to the development of MSC-based treatment for cGVHD (160, 161). NK cells may also target APCs (162), which are important drivers of cGVHD, as described in the following chapter. This APC-killing effect is potentiated in the presence of activating KIR2DS1 receptor binding to HLA-C2 on APC surface (163). Another receptor playing a role in this interaction may be NKp46, which binds to ligands present on DCs, and absence of NKp46 on NK cells leads to significantly increased GVHD (164, 165). NK cell degranulation towards activated T cells was decreased when either NKG2D, NKp46, or DNAM-1 was blocked, and decreased degranulation was associated with increased risk for cGVHD and aGVHD (166). However, the beneficial role of cytotoxic NK cell activity may depend on the disease stage, as patients with late aGVHD and cGVHD have increased numbers CD56dim cytolytic NK cells (75). Whereas NK cell cytotoxic activity can protect against GVHD development, it was shown that NK cells producing pro-inflammatory cytokines can induce it. A GVHD-stimulating effect of IFN-γ and TNF-α production by NK cells contributed to GVHD in both mice and humans (167, 168).

### NK cell reconstitution dynamics impact cGVHD development

Due to their rapid reconstitution after HSCT (117), NK cell count reaches its normal numbers after one month as compared to T cells, whose numbers are usually still low three months post-transplant (117, 169). However, the circulating NK cells as late as three months post-transplant are mostly of the CD56bright subset, constituting nearly 40% of all NK cells in the transplanted patient, compared to only 10-15% in healthy individuals (whose NK cells are mostly of the CD56^dim^ subset) (170). CD56^bright^ NK cells are characterized by high expression of the inhibitory receptor NKG2A. CD56^bright^ cells in post-transplant patients are also reported to have unusually impaired IFN-γ secretion (169, 171).

Presence of T cells in the graft seems to have a positive effect on reconstituted NK cells and their development, as it increases their cytokine production and cytotoxicity (167, 172). However, both cellular subsets seem to have a different effect on cGVHD development, as e.g. donor-derived naïve T cells can increase the risk of cGVHD (47). While T cells can exacerbate GVHD, NK cells are able to suppress it. Low reconstitution levels of NK cells were linked to a poorer outcome in cGVHD patients (173) and low numbers of reconstituting NK cells as well as a dysfunctional phenotype is associated with aGVHD relapse in patients receiving PTCy (174). One of the possible mechanisms behind this is lysis of alloreactive T cells, mediated by NK cell receptor NKG2D. This process leads to killing by induction of FasL-mediated apoptosis and degranulation. This occurs possibly due to increased expression of an NKG2D ligand on alloreactive T cells during aGVHD, as well as trafficking of NK cells to GVHD-affected tissues driven by chemokine release (175, 176). However, this mechanism has not been verified specifically in cGVHD patients as of yet. Interestingly, a study on mice showed that even residual recipient NK cells could prevent aGVHD by targeting T cells in a minor antigen mismatch setting (177). The CD56^bright^ NK cells were observed to be decreased in patients developing cGVHD (46, 178–180). Lauener et al. demonstrated that HSCT patients later developing cGVHD already displayed with lower levels of CD56^bright^ cells at day 100 compared to GVHD-free HSCT patients (181), suggesting a causal involvement of this subset in cGVHD. However, it is so far unclear what causes the loss of CD56^bright^ cells in patients prior to cGVHD and how this could be prevented. Interestingly, patients receiving PTCy have delayed T and NK cell reconstitution with a higher proportion of immature CD56^bright^ CD16^-^ NK cells compared to patients receiving conventional GVHD prophylaxis (174, 182). In a cohort of haplo-HSCT transplanted an increased ratio of CD56^bright^CD16^-^ immature NK cells to CD56^bright^CD16^+^ mature NK cells was correlated with the development of cGVHD (182), suggesting that the protective effect of the CD56^bright^ population depends on the maturation status. A higher proportion of CD56^bright^ cells is also partly responsible for the therapeutic effects of extracorporeal photopheresis (ECP) treatment in cGVHD (183). The protective effect of CD56^bright^ NK cells may be due to their ability to suppress effector T cells in a contact-dependent manner (184). This may be mediated by NCR receptors NKp44 and NKp46, although not all studies support this (181, 184). This regulatory NK cell subset was also described to be perforin^+^, Granzyme B^+^ and NKp46 +  (181).

### NK-cell derived EVs can polarize T cells

Activated NK cell-derived EVs (NK-EV) also have potential for a direct effect on cells, whereby EVs containing cytotoxic molecules such as perforin or FasL (185) have been shown to exhibit antitumoral effects. However, NK-EV miRNA have also been demonstrated to play a functional role in the polarization of a CD4^+^ T cell response. EVs containing miR-10b-5p and miR-92a-3p were shown to downregulate GATA3 mRNA, the Th2 signature transcription factor; resulting in an increase of Th1-like cells producing Interferon gamma IFN-γ, and Interleukin-2 *in vitro* (186). *In vivo* this would exacerbate or sustain the pro-inflammatory shift seen in cGVHD.

The dual role of NK cells *via* cytotoxicity and production of pro-inflammatory cytokines, as well as complicated interactions between T cells and reconstituted NK cells, may be responsible for discrepant results of studies on NK cell in human GVHD development. It is hypothesized that significant pre-activation of NK cells can lead to their GVHD-inducing character to prevail over their T cell killing capabilities (120). Although the role of NK cells in GVHD is not entirely clear, many studies have reported that early reconstitution of NK cells is associated with favorable outcomes, suggesting that by maximizing NK cell functions, HSCT outcomes could be improved (187).

## Antigen presenting cells as disease drivers in cGVHD

**Figure 4:**
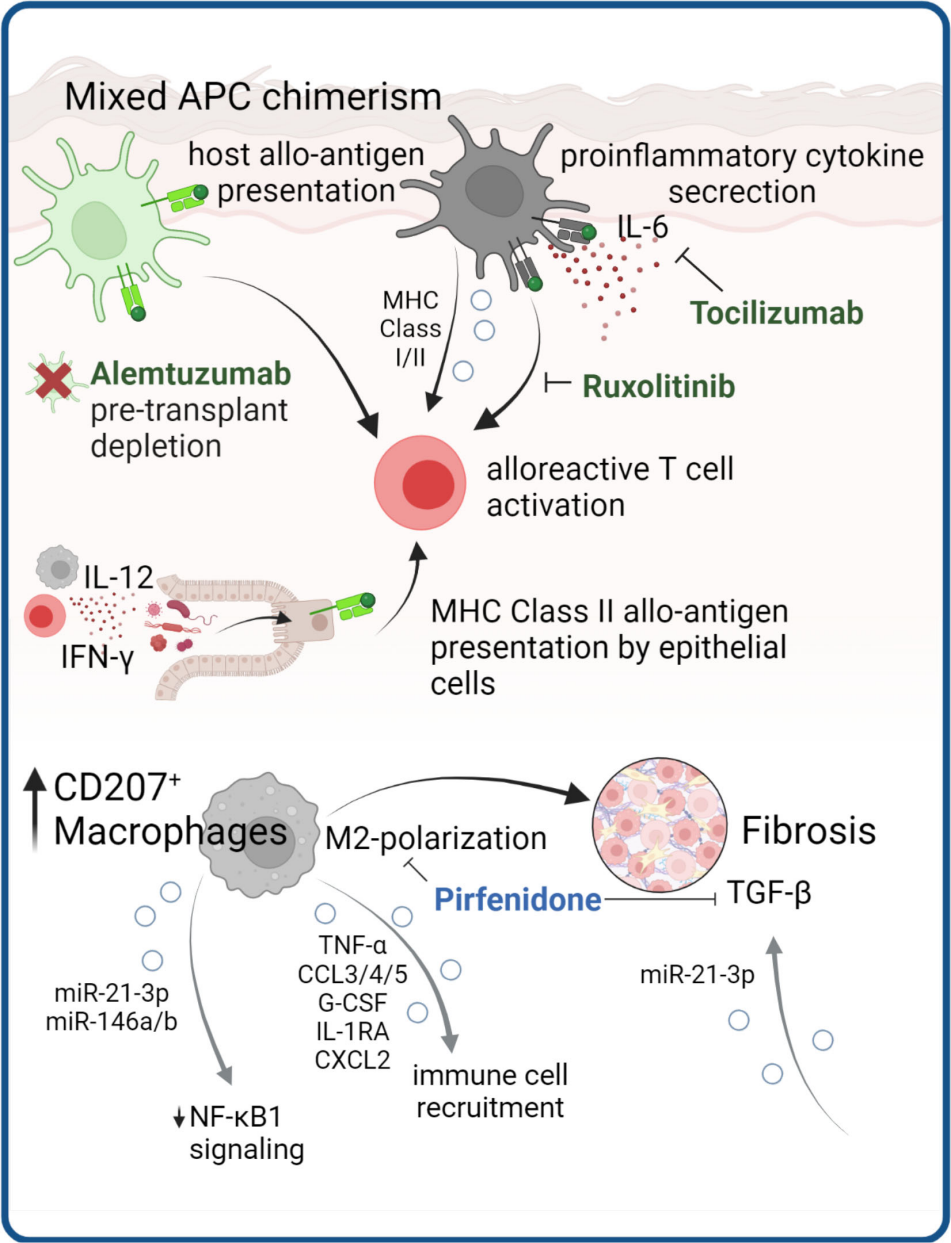
APCs as cGVHD disease drivers. APC are instrumental in cGVHD development through presentation of allo-antigens to T cells and production of pro-inflammatory cytokines. Antibodies targeting these mechanisms are in clinical use (in green). Structural cells, such as epithelial cells can obtain MHC class II antigen-presenting properties presenting allo-antigen in the gut of mice. In cGVHD macrophages with M2 polarization are expanded and contribute to tissue fibrosis, which in mouse models could be ameliorated by pirfenidone. There are several lines of evidence that EV release by APC mediate processes implicated in cGVHD pathology.

Professional APCs are a heterogeneous group of immune cells, that are highly efficient in processing and presenting antigens to promote an adaptive immune response. Major populations of APCs in human tissues include DCs, macrophages and - to a lesser extent - cells of the B-lineage. Together with co-stimulatory signals upon formation of an immunological synapse, APC may activate CD4^+^ T cells by exogenous antigen presented *via* MHC-II, and cytotoxic T cells *via* presentation of endogenous antigen on MHC-I (**Figure 4**).

### Host and donor APC can initiate pathologic T cell responses in acute and chronic GVHD

In the context of HSCT, host (tissue) antigens can be presented by host APCs both *via* MHC-I and MHC-II or, indirectly, cross-presented by donor APCs *via* MHC-I (188–191). With this in mind, several studies around the turn of the millennium have focused on investigating the presence of resident host APCs that survive conditioning and have the ability to initiate GVHD. Interestingly, during pre-transplant conditioning, the APC compartment of barrier tissues remains fairly unaffected, and replacement with donor APCs after HSCT is incomplete (192–194). Together with the presence of remaining recipient tissue-resident T cells (51, 52), this results in mixed chimerism of both APC and lymphocyte populations within tissues after HSCT. Indeed, due to their capacity to present host allo-antigens, host APCs are required for initiation of GVHD responses in tissues (195, 196). However, the majority of APCs in GVHD lesions were found to be of donor origin (197, 198), suggesting a prominent role for host APCs in the propagation of inflammation by cytotoxic T cell activation.

In consequence, preventive and therapeutic strategies for GVHD have focused both on depletion of host APCs, for example by pre-transplant conditioning regimens containing alemtuzumab (CAMPATH) (199), and the modulation of donor APC cytokine responses, including IL-6-blockade with tocilizumab (200). We have above discussed the effects of Ruxolitinib on pathogenic T cell signaling, however this drug also can modulate APC function. Ruxolitinib impairs human DC activation and DC-mediated T cell activation (201), thereby additionally benefiting therapeutic effects.

Regarding specific APC types in GVHD inflammation, there is a redundancy as to which cells can initiate GVHD. In skin, host Langerhans cells are potent activators of donor cytotoxic T cells (202) and cutaneous aGVHD results in replacement by donor LCs, suggesting activation and migration (194). However, LC are not essential to murine GVHD and in the absence of LCs, other APC subsets may drive inflammation (203).

In murine models of cutaneous and pulmonary cGVHD the infiltration of M2-polarized donor F4/80^+^ macrophages preceded disease development and blocking this infiltration with anti-CSF-1R antibody significantly reduced cGVHD in both organs (198). Expansion of myeloid cells expressing the type-2 macrophage marker CD163 in steroid refractory aGVHD (204), and CD207^+^ donor-derived macrophages in cGVHD-affected tissues (198), suggest overall M2-polarization of GVHD macrophages. Although macrophage polarization profiles have not been formally investigated in GVHD subtypes in humans, the high plasticity of macrophages to exert both pro-inflammatory and pro-fibrotic functions could indicate a role in non-classical GVHD subtypes. Of note, Adams et al. recently demonstrated that BM-derived MHC class I^I+^ macrophages are critical mediators of neuroinflammation in a cGVHD mouse model (205). During murine skin aGVHD, a distinct donor-derived macrophage population expands, and maintains a proinflammatory phenotype even after GVHD resolution, leading to a loss of tolerance and Treg suppressive capacity, which might further prime chronic inflammation and fibrosis (206). Du et al. treated mice with cGVHD with the antifibrotic small molecule Pirfenidone, which was FDA-approved for treating idiopathic pulmonary fibrosis. This resulted in reduced donor BM-derived M2 macrophage infiltration, decreased expression of pro-fibrotic TGF-beta, and reduced lung fibrosis (207).

Compared to macrophages, DCs show more rapid engraftment and increased donor chimerism after HSCT (208). Common DC subsets in human tissues include conventional DCs (cDCs) and plasmacytoid DCs (pDCs) (209). While cDC were shown to be a critical donor APC presenting allo-antigen after HSCT (210), recovery of donor pDCs early after HSCT reduces GVHD (211, 212).

### Antigen presentation by non-professional APC contributes to inflammation

Finally, more recently, non-hematopoietic cells have come into focus as cells that can develop antigen-presenting ability under specific conditions. This includes keratinocytes (213), fibroblasts (214), alveolar epithelial cells and intestinal epithelial cells, the latter of which were found to initiate lethal gastrointestinal GVHD by MHC class II-presentation of allo-antigens (215). Koyama et al. demonstrate in a mouse model of GI-GVHD that IL-12 from ileal macrophages, IFN-γ from lymphocytes, as well as sensing of microbiota-derived TLR ligands induces MHC-II upregulation on intestinal epithelial cells, which then present allo-antigens to donor T cells to induce lethal GVHD (215). This study already highlights the complex interactions with the microbiome in GVHD target organs at barrier sites, which will be discussed in the next chapter.

Apart from antigen presentation, non-hematopoietic cells, specifically fibroblasts are involved in cGVHD pathogenesis as main executors of tissue fibrosis by excessive production of extracellular matrix and collagen deposition in response to chronic inflammation. Interestingly, a recent report demonstrates that fibrosis could by amplified through EV signaling. Dermal fibroblasts that were exposed to cGVHD patient derived EVs displayed significantly higher levels of fibrosis mediator TGF-β compared to healthy EV exposure (216). Another preliminary study in a small cohort of cGVHD patients found significantly increased miRNA miR-29-3p in patient EVs, which was predicted to regulate hub genes involved in TGF- β signaling and fibrotic processes, such as type I collagens or MMP2 (217).

### EVs regulate antigen presentation

EVs also play a key role in antigen presentation, whereby EVs released by APCs carry surface MHC Class-I and MHC Class-II molecules and can directly stimulate CD8^+^ and CD4^+^ T cells, respectively (218, 219). The capacity of EVs to stimulate T cells, including naïve T cells, can be enhanced by their interaction with DCs. The presence of specific adhesion molecules that are involved in EV binding to DCs, such as integrins and ICAM1, depends on the lineage and activation stage of the parent cells (220). Indeed, EVs released by LPS-treated DCs bear more MHC Class-II and exhibit a more potent T-cell stimulatory function than EVs secreted by immature DCs. Indeed, mature DC EVs transfer the ability to activate naïve T cells to non-professional APCs (221, 222). A percentage of the acquired EVs remain on the surface of the target cell, whereas the rest are internalized by phagocytosis or micropinocytosis. Immature DCs internalize EVs more efficiently than mature DCs, whereas mature DCs retain more EVs on the cell surface (223). Once internalized by DCs, the EV-derived peptide-MHC (p-MHC) complexes can be degraded by the APCs and used as a source of peptide to interact indirectly with T-cells. p-MHC complexes of EVs attached (or fused) to APC surfaces can also be presented directly to T-cells without the need for p-MHC reprocessing, through a mechanism known as cross-presentation (222). The finding that optimal T-cell stimulation occurs when EVs transfer p-MHC complexes to mature DCs indicates that the acceptor APCs provide the required costimulatory molecules that are absent in the EVs (224). There are several lines of evidence suggesting that, following allograft transplantation, cross-presentation of recipient’s APCs with donor MHC molecules could be mediated through EV transfer (225).

Professional and non-professional antigen presenting cells were shown to provide either pro- or anti-inflammatory functions in GVHD, depending on the specific cell type and context. Notably, APC can drive GVHD using different mechanisms including direct priming of T cells, release of EV and contribution to the cytokine milieu. As such, APC are promising targets in therapeutic modulation of T cell responses in GVHD.

## Microbiome-mediated effects in cGVHD

**Figure 5:**
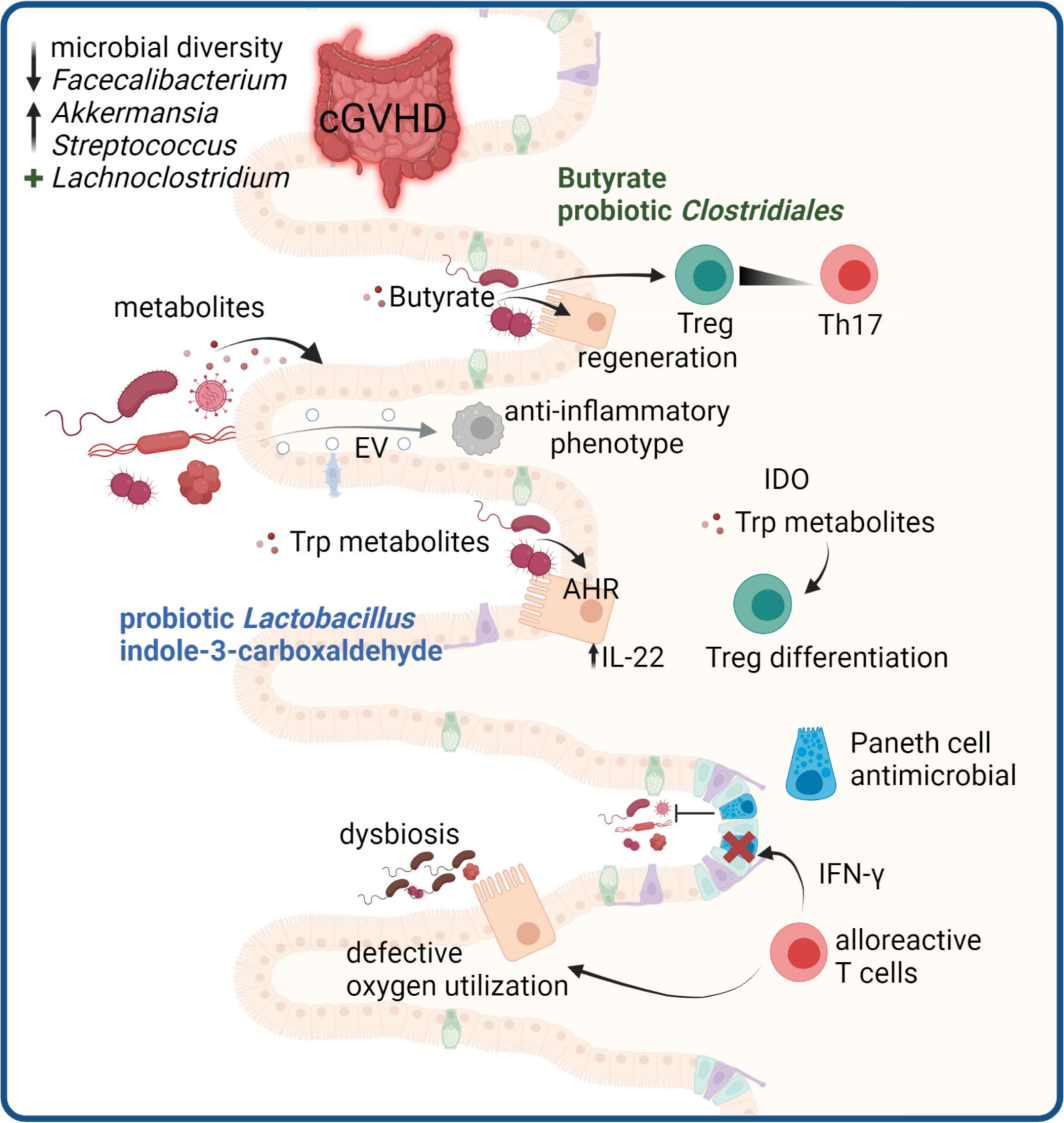
Microbiome-mediated effects in cGVHD. GVHD development in the gut is associated with a disturbed microbiome. In cGVHD decreased microbial diversity with reduced *Faecalibacterium* and increased *Akkermansia* and Streptococcus species are reported, whereas high abundance of *Lachnoclostridium* is protective. Microbial metabolites are critical mediators of gut immune responses. Butyrate promotes Treg and regeneration of epithelial cells. Trp metabolites derived from microbes can protect the epithelium via IL-22 induction and microbial or tissue cell derived Trp metabolites promote Treg differentiation. Paneth cells in the gut produce antimicrobial peptides, and are targets of destruction by alloreactive T cells, leading to impaired host defense. Damaged epithelial cells have defective oxygen utilization, leading to loss of physiologic hypoxia in GVHD which drives microbial dysbiosis. Microbiota can release EV that modulate host immune responses.

The gut microbiome is defined as the genetic and functional profile of the microbes residing in the gut. Together, these microbes encode over three million genes, providing a much wider variety and flexibility than the 23,000 protein-coding genes of the human genome (226, 227). The microbiota have gained increasing attention for its influence on human health and disease, and has been shown to influence the epithelial mucus layer, promote development of lymphoid structures, function in activation and differentiation of lymphocyte populations and balance the production of IgA and antimicrobial peptides (228) (**Figure 5**).

### Mycobiome dysbiosis occurs after allo-HSCT

The microbiome comprises bacteria, fungi and viruses, however in the context of GVHD studies mostly focus on the bacterial microbiome. We are only beginning to understand the role of the mycobiome in aGVHD. In a retrospective study of 153 allo-HSCT recipients, patients with intestinal *Candida spp* colonization showed significantly more grade II-IV aGVHD (229). Rolling et al. profiled mycobiota dynamics in allo-HSCT patients and found stable overall fungal diversity, but high day to day variance. Interestingly, stable expansion of *Candida parapsilosis* complex species in fungal cultures associated with higher transplant related mortality (230). It remains to be determined if there is causal role for fungi in the development or promotion of aGVHD and cGVHD.

### Gut microbiota composition influences GVHD development

The bacterial microbiome has emerged as a key player in GVHD development and progression. It has long been postulated that germ-free mice have prolonged survival compared to conventional mice after allo-BMT (231, 232). However, this was recently challenged by a study showing that after allo-BMT, germ free mice displayed greater GVHD then age-matched littermates kept in specific pathogen free conditions, indicating that the microbiome itself can mitigate GVHD (233). There is a plethora of studies demonstrating that the gut microbiota, through their metabolites, are able to influence host immune responses, and that loss or gain of certain bacterial taxa may influence GVHD pathology. The importance for microbiota on GVHD development is covered in detail in recent excellent reviews (234, 235).The composition of the microbiome at the time of transplant or post-transplant can impact the outcome and risk for developing aGVHD and cGVHD. Numerous studies have shown that the severity of GVHD after allo-HSCT is associated with decreased microbial diversity of the GI tract, with a particular loss of commensal anaerobes (236–238). Seike et al. recently discovered that dysbiosis in the gut of allo-BMT mice is a consequence of a defective oxygen utilization by intestinal epithelial cells, caused by pathogenic T cell attack, leading to a loss of physiologic hypoxia (233). In this model, dysbiosis before transplantation did not affect GI-GVHD severity, arguing that dysbiosis is more a consequence than a cause for GI-GVHD. In a case-control study with collected stool samples, Markey et al. showed that a reduction in *Faecalibacterium* and an increase in *Akkermansia* and *Streptococcus* was associated with development of cGVHD (239). In the same study, increased abundance of *Lachnoclostridium* and Clostridium at day +100 after transplantation was associated with better prognosis and cGvHD-free survival (239). For aGVHD, several studies have indicated that increased abundance of *Lachnospiraceae* is associated to reduced risk and increased overall survival of aGVHD (238, 240, 241). It is not well understood yet how GVDH prophylaxis with PTCy affects the microbiome, however cyclophosphamide-microbiome interactions have been studied in the context of cancer. In antibiotic treated mice the anti-cancer activity of cyclophosphamide was reduced, suggesting a crucial involvement of the microbiome in the immunomodulatory activity of the drug (242). Dallière et al. demonstrated that in mice *Enterococcus hirae* translocate to secondary lymphoid organs during cyclophosphamide treatment, leading to an increase in intratumoral CD8^+^:Treg ratios, while *Barnesiella intestihominis* facilitates the infiltration of IFN-γ producing γδ T cells (243). How dysbiosis in HSCT patients is affected by PTCy and whether there are any implications for host immune responses and GVHD pathobiology must be addressed in future studies.

### Microbial metabolites affect the epithelium and host immunity

The commensal bacteria influence host immunity through direct interactions with the epithelium, or *via* metabolites. Short chain fatty acids produced by anaerobic bacterial fermentation of dietary fibers in the colon have been implicated in mediating epithelial layer integrity and optimal immune cell homeostasis in the gut. Of these, butyrate produced by Firmicutes such as *Clostridia*, modulate immune cell phenotypes and composition, and in particular the skewing towards Tregs at the expense of Th17 (244). This has important implications, as an over-weight of Th17 cells can drive chronic GI GVHD due to production of profibrotic cytokines (245). Butyrate also contributes to epithelial cell regeneration. A study by Mathewson et al, demonstrated that administration of butyrate or a probiotic cocktail of 17 Clostridiales species to allo-BMT mice led to amelioration of GVHD through improved epithelial layer integrity (246). Similarly, administration of selective antibiotics that preserve the presence of butyrate-producing bacteria lead to reduced GVHD mortality in allo-HSCT patients (247). In line with these finding, a lower plasma concentration of butyrate and propionate on day 100 was observed in patients that developed cGVHD (239). However, there is conflicting data as another study has shown abundance of butyrate-producing bacteria in patients developing steroid-refractory cGVHD (248).

Among other metabolites, the essential amino acid tryptophan is metabolized to kynurenin by the enzyme indoleamine 2,3-dioxygenase (IDO), expressed in intestinal epithelial cells and immune cells. A significant proportion of tryptophane is also metabolized to tryptamine and indole metabolites through tryptophanase and decarboxylase enzymes, *via* the *Lactobacillus* and *Clostridia* genus (249). Tryptophane metabolites interact with the nuclear transcription factor aryl hydrocarbon receptor, which leads to increased production of the epithelial protective cytokine IL-22 by ILC3s (250). Probiotic administration of *Lactobacillus* was shown to reduce GVHD in a mouse model (236), and another mouse study showed that oral administration of the indole metabolite indole-3-carboxaldehyde reduced gut epithelial damage and reduced overall GVHD pathology and mortality (251). The mechanism was demonstrated to involve *Lactobacillus* and ILC3s (252). In addition, tryptophane metabolism through IDO also promotes Treg differentiation (253), and has been associated with suppression of GVHD in animal models (254).

Maintenance of an optimal bacterial diversity is governed by multiple external and intrinsic factors. Within intestinal crypts, specialized cells with antimicrobial functions, the Paneth cells, play a role in maintaining the homeostatic balance between host and the microbiome. Upon pre-conditioning prior to allo-HSCT, the epithelium may be damaged, and loss of crypt structures has been associated to delayed recovery from GVHD and steroid-refractory GVHD (255–257). The disruption of crypts is accompanied by fewer Paneth cells, that produce essential anti-microbial peptides and growth factors necessary for maintenance of intestinal stem cells. Crypt destruction was recently demonstrated to be mediated in an IFN-γ-dependent mechanism by alloreactive T cells that invade the crypt structures early after transplantation (258, 259).

### Microbiota-derived EVs regulate host immunity

The immune modulating effects of EVs are not constrained to self-cells. Microbiota have also been found to excrete EVs, which are more akin to shedding microvesicles. These outer-inner membrane vesicles (O-IMVs) have been described by Pérez-Cruz et al., which in their study derived from several pathogenic Gram-negative bacteria and contain cytoplasmic components (260). Like EVs, these particles display and encompass host cell proteins and nucleic acids, which are known to elicit an immune response (260). Contrarily, EVs produced by beneficial microbes such as *Clostridium butyricum* polarize macrophages into an anti-inflammatory phenotype, aiding in treatment and remission of a murine colitis model (261). Though the potential exists for anti-inflammatory O-IMVs, the dysregulation of the various microbiomes as a result of cGVHD may shift towards pathogenic microbes and exacerbate disease. Further research is necessary to investigate these effects in pre-clinical models and patients.

The described studies highlight the potential of manipulating the bacterial composition or metabolites to not only promote epithelial regeneration, but to also promote an immune cell composition that may ameliorate and prevent the damage caused by alloreactive donor T cells, thus indicating that manipulation of bacterial metabolites can be exploited clinically. Therapeutic strategies in terms of cGVHD should involve prebiotics or probiotics that skew the balance in favor of bacterial strains that provide beneficial metabolites and out-compete strains with an unfavorable impact. Alternatively, the use of fecal transplantations, that has been shown to ameliorate GVHD in patients with steroid-refractory aGVHD (262), may be considered.

## Discussion

### Understanding cellular networks within cGVHD-affected tissue will pave the way forward to apply precision medicine

In this review we summarized current knowledge about disease mediating signaling in different cell types involved in the pathogenesis of cGVHD. Disease complexity and heterogeneity is reflected by the involvement of different cellular players described here, displaying multiple options for inter-and intracellular signaling that can either promote or inhibit cGVHD. While compartmentalization of the immune system into different cell types within specific organs is useful study single mechanisms, in disease all the described pathways can be active in the given tissue and organism at the same time. The difficulty lies in the deconvolution of these signaling networks, to find targets for effective preventive and therapeutic treatments.

A lot of our knowledge about cellular disease mechanisms comes from mouse studies. With the advance of (spatial) single-cell technologies we are gaining a better understanding of human tissue- and disease specific pathways driving certain phenotypes (263–266). However, there is high variability between patients, and at present markers to predict therapy response or disease outcome are lacking.

In the age of precision medicine, we require reliable biomarkers for patient stratification to determine the best possible treatment for every individual patient. The collection of large-scale open-source OMICs data from patient cohorts all over the globe is increasing, as is the development of sophisticated bioinformatic tools to predict cell-cell communication and tissue organization in homeostasis and inflammation (267–271). With the rapid improvement of deep learning methods, biologists and clinicians have the tools at hand to better understand cGVHD pathology from a systems perspective and uncover disease-associated processes that have high therapeutic potential and can be tailored to the patients’ specific immune status (272).

As lately discussed in the NIH Consensus document on personalized treatment for cGVHD (273), it will be crucial to understand the connection between clinical effects and the underlying biological mechanisms in cGVHD therapies currently in use, which will also allow the development of new, refined therapeutic approaches. Importantly, the authors emphasize the need for conducting clinical trials for first line treatment without the concomitant use of glucocorticoids, which hamper the investigation of disease-modulating effects of the used therapeutics in those trials (273). Close monitoring of patients during treatment with intensive sample collection and biological analysis will be necessary to improve our understanding of cGVHD pathogenesis, and definition of reliable biomarkers which can be used to design personalized treatment schemes (273). Ultimately, a better understanding of immune cellular crosstalk and its regulation in cGVHD will lead to the development of better drugs and therapies and improve the life of patients.

### Studying cellular crosstalk *via* EVs is a new way forward in cGVHD diagnosis, patient stratification and development of therapeutics

Collectively, the studies highlighted in this review demonstrate that the activities of EVs can affect wide ranging immunoregulatory functions on a variety of immune cells which are known to be involved in the etiology of GVHD, including modulating antigen presentation, immune activation, immune suppression, immune surveillance, and intercellular communication, *via* both direct and indirect mechanisms. Given the ubiquitous involvement of EVs in immunomodulation, their translational potential as biologically relevant biomarkers and as novel therapeutics offers an exciting area for future discovery in the GVHD field. Despite this however, many questions remain unanswered and will be the focus of immediate research, particularly to decipher their precise roles in post-HSCT immune cell crosstalk and regulation. As a field in its relative infancy, fundamental questions remain about the best way to isolate, characterize and study EVs, including the effect of variation in sample collection, storage protocols and cell manipulation on EV productivity and molecular cargo. This in turn influences comparability between GvHD studies, which are already made challenging due to the heterogeneity in sample cohorts in terms of HSCT conditioning, prophylaxis and GvHD therapies. Many of these questions around best standardization in EV research are the topic of intense scrutiny by the International Society of Extracellular Vesicle (ISEV) specialist task force groups. Moving towards future EV research, there will be much focus on the heterogeneity of EV populations themselves, including those produced by single immune cell types, as well as the variation in EV populations in the circulation and how these can be traced back to immune cell of origin. Given the key advancements in our understanding of the role of EVs in immune cell crosstalk, as well as the complexity of the immune system, immune cell derived EVs look to be an area of intense research and development in future years.

## Author contributions

GS and LMG conceptualized the manuscript. LMG, KJS, PŁ, JS, SJB, ESU, KB-K, HTG, REC, MI, and GS wrote the manuscript. LMG drafted figures and edited the manuscript. All authors contributed to the article and approved the submitted version.
